# Co-creating a process of user involvement and shared decision-making in coordinated care planning with users and caregivers in social services

**DOI:** 10.1080/17482631.2020.1812270

**Published:** 2020-09-17

**Authors:** Ola Knutsson, Ulla-Karin Schön

**Affiliations:** a Department of Computer and Systems Sciences, Stockholm University, Stockholm, Sweden; b Department of Social Work, Stockholm University, Stockholm, Sweden

**Keywords:** Shared decision-making, social services, future workshops, mental disabilities, coordinated care planning

## Abstract

**Purpose:**

Although user participation and shared decision-making in formal statutory coordinated care planning are described as central, they remain to be implemented. The aim of this study is to explore how collaboration and shared decision-making in the social services can be realized in formal care planning activities with people with mental disabilities.

**Methods:**

We conducted eight workshops with 12 users and 17 caregivers to investigate existing barriers to and possible solutions for participation in coordinated care planning.

**Results:**

Workshop formats and techniques from participatory design generated rich research materials illustrating challenges currently experienced by users and caregivers in care planning work, as well as a large variety of solutions to these challenges. They also illustrated differences in how participation is understood and the conditions required to realize shared decision-making between users and caregivers.

**Conclusions:**

An improved coordinated individual plan (CIP) process emerged, based on the active participation of users and caregivers. This process is a familiar and transparent process for users and caregivers, reflecting the needs and preferences of users at all stages. It requires careful preparation and collaboration with the users, as well as caregiver flexibility.

## Introduction

In this study we held a series of workshops to explore how collaboration and shared decision-making (SDM) can be applied in the social services when developing coordinated care plans with people with mental disabilities. Despite a strong emphasis on user involvement and SDM in care planning in social services, the scientific knowledge is currently limited on *how* to achieve these goals (McCormack & McCane, 2011; Nykänen, 2019). The concept of user involvement is sometimes described as the most opaque of terms and there are several degrees, or levels, of client participation that need to be distinguished (Levin et al., 2017). Participation can range from the user as an information provider, to them being in charge of service provision (Julkunen & Heikkilä, 2012). User involvement in social work decisions, for example, coordinated care planning, involves the opportunity for the user to provide real input into activities, information and support services (Eriksson, 2015; Sandman & Munthe, 2010). User participation has been described to include principles of respect for the knowledge of both the user and the caregivers, thus creating collaborative processes and personalized services (McLaughlin, 2009). User participation in social services challenges traditional paternalistic models of decision-making, where caregivers make decisions they believe lie in the best interests of the client (Grim et al., 2019). User involvement in social work decisions derives from the belief that user knowledge is indispensable when reaching effective decisions about a client’s difficulties and solutions (Levin et al., 2017; McLaughlin, 2009). Current decision-making in social services has been criticized for abuse of power and for excluding the users’ right to be informed and consulted (Nykänen, 2019). Underestimating the importance of user experience and participation may affect the suitability of the offered interventions and care planning in general (Schön et al., 2018; Shier, 2001).

Shared decision-making is an interactive process between the user and the caregiver that aims to increase the client’s participation in decision-making, where both user and caregiver participate actively (Davidson et al., 2017). This means that clients are provided with information and options for a joint decision to be made. Shared decision-making is an evidence-based approach that allows users and caregivers to make informed decisions together, taking into account both the users’ and the caregivers’ knowledge and scientific facts (Stovell et al., 2016), to meet the users’ need for involvement in and influence over their own care. It is a collaborative process that creates the conditions for users and caregivers to create goals and objectives of the care efforts together, and make informed decisions (Elwyn et al., 2013; Hamann et al., 2010). Based on models of participation such as Arnstein’s ladder of participation (1969), later developed by both Shier (2001) and Hart (1992), SDM creates a system of collaboration and changes the client’s role from passive participation to active involvement. However, Arnstein has been criticized for a unilateral focus on power without including the fact that the ability of social service clients to participate may vary (Treichler & Spaulding, 2017). When the focus is merely on the power aspect, participation aspects may be neglected, despite the fact that involvement may have a value in itself (Tritter & McCallum, 2006). Naturally, clients and social workers can never be on an equal footing when decisions are made in social services, since the exercise of authority is embedded in the role of the social worker. The professional role comprises making decisions based on given laws and guidelines. Still, the opportunity for equal planning and informed decision-making is emphasized (Levin et al., 2017; Schön et al., 2018).

In Sweden, as in many other countries, SDM has been advocated as an important method for strengthening person-centred care and social practice (National Board of Health and Welfare, 2011, 2018) but so far, it has received relatively little attention in social services, although studies show that users would like to be involved in planning and decision-making (Grim et al., 2016). International studies on SDM report a significant impact of users’ involvement on their commitment and satisfaction, as well as an increased knowledge about disability and a greater perceived participation in decisions (Davidson et al., 2017; Duncan et al., 2008; Hamann et al., 2010; Stovell et al., 2016). Most social work clients have the ability and the desire to participate in decisions about their care (Grim et al., 2016, 2019; Hamann et al., 2010), but the desire for participation is still greater than the degree of participation in practice (Grim et al., 2016; Torrey & Drake, 2011).

To explain the reason for this gap, research has pointed to a number of barriers to a successful implementation of SDM, such as time constraints, the excessive workload of caregivers, a lack of training for caregivers and users, and a lack of access to information and decision-making support for users (Duncan et al., 2008; Schön et al., 2018). Research also shows that, in order for SDM to be successfully implemented in social services, caregivers and users need knowledge and the willingness to practice the method, needs to be supported’ (Hamann et al., 2010; Levin et al., 2017). Successful implementation is therefore about *attitudes* to creating participation and utilizing client knowledge and preferences, but also about the *ability* to work with the method (Schön et al., 2018). It is common for users to experience uncertainty about their own knowledge and take on a passive role as recipients of the knowledge and skills of the providers (Duncan et al., 2008; Grim et al., 2016). The knowledge about how SDM can be implemented in these constrained social services is limited, as is the knowledge about how trust in the caregivers’ own decision-making competence and, in particular, the clients’ decision-making and ability to participate in decision-making can be enhanced. It has been shown that a traditional, top-down implementation of SDM in social services has a limited impact (Levin et al., 2017; Schön et al., 2018). However, increased knowledge about contextual conditions and about how clients and caregivers believe that SDM can be implemented in their specific context may benefit future implementation of SDM.

An area where participation and SDM have been highlighted in Sweden is in connection with the development of coordinated individual plans (CIPs). A CIP is created when users are in need of long-term support from more than one care and support provider, such as social services and psychiatric substance abuse treatment. In Sweden, there is a requirement to carry out a CIP for people in need of support from both social services and health care. This is regulated by law, to assure users that individual needs will be met. The process of developing CIPs needs to be guided by the principles of SDM, in order to promote user participation and collaboration (Nykänen, 2019). However, although user participation in such care planning is described as central, it still remains to be realized (Nykänen, 2019; Schön et al., 2018).

Table I illustrates the five steps in creating a CIP (SKL, Sveriges Kommuner och Landsting, 2019):

**Table I.** The five stages of creating a coordinated individual plan (CIP).

Stage	Content
1. Introduction	Creating a mutual agenda; describing roles and exploring whom to invite; and obtaining consent.
2. Choice & options talk	Informed discussion about choices and treatment options.
3. Invitation to a meeting	The meeting is to follow the agenda created by the user and caregivers.
4. Meeting	Decision-making skills and authority in user and caregivers: making informed decisions and creating a CIP.
5. Follow-up	Joint action plan.

This study intends to explore the experience of users and caregivers regarding how collaboration and SDM can permeate the CIP development process. Each of the stages above have been explored through workshops (see below).

### Aim

The aim of this study is to explore how collaboration and SDM can be realized in coordinated care planning processes and decision-making in social services—and specifically, how increased participation in coordinated care planning can be facilitated for users with mental disabilities.

## Method

To explore how the process of creating a CIP, based on SDM, could be designed, we applied workshop formats and techniques from participatory design (Muller, 2007). Participatory design is not one single method, but a diverse collection of methods, techniques and tools to involve users in design processes. Even more importantly, the approach intends to equalize power relationships, develop democratic practices focusing on people’s everyday situations, and create mutual learning and accessible tools and techniques for participation (Kensing & Greenbaum, 2013). The design in this case entails the design of the CIP process itself including the communication and interaction between people involved in the process.

The chosen participatory approach had two goals. The first was to collect data from the workshops about perceived difficulties in the CIP process and possible solutions using different techniques. The second was to achieve a design (or several designs) for an improved CIP process, based on the active participation of the users and caregivers (e.g., by telling, making and enacting) during the workshops (Brandt et al., 2013), framed by improved collaboration and SDM. Two types of workshops were held: *Future Workshops* and *Present and Future Stories Workshops*, and four workshops of each type were conducted. The purpose, methods and participants in these workshops are described below.

### Future workshops

Future Workshops are based on the work of Jungk and Müllert (1984), and the purpose is to give a voice in different processes to people affected by a decision but often excluded from the decision-making process. The purpose of a Future Workshop is to identify the obstacles that a group of people may encounter concerning a specific issue and to find new solutions to these obstacles together. A Future Workshop aims to combine intuitive processes (brainstorming) with more analytical ones (clustering the results of the brainstorming) (Jungk and Müllert, 1984). The main method used in a Future Workshop is brainstorming and putting down short sentences or key words on post-it notes.

The Future Workshops in this study were limited to 2 hours each because of the busy work schedules of the caregivers. In addition, workshops of over 2 hours were considered to be too demanding for the service users. Because of the time restrictions, our focus was on three phases. The first of these was the *critique phase*, or *problem-finding phase*. With the aim of acquiring knowledge of the current situation concerning CIPs (How does the process work today? What are the problems?), during the critique phase the participants were asked “What comes to mind when you read these questions?” and instructed to write down problems in a brainstorming session.

A quick analysis of the criticism/problem-finding phase was made by the workshop participants, and this formed the basis of the next phase, the *fantasy phase*. The fantasy phase had the aim of finding solutions to the problems identified in the analysis of the problem-finding phase. Brainstorming was used to come up with numerous solutions to the problems identified, without any restrictions on solutions.

The final phase, termed *implementation phase*, included selecting from the proposed solutions, and refining those that the participants considered realistic to develop further.

The workshop started with a short introduction to CIP work in the shape of a 3-minute film made by the authorities (SKR), to create a common ground regarding the optimal CIP process. The film was followed up with a question about the film and whether it presented the participants’ experiences of CIP processes. It was decided that the workshop should solely focus on the CIP process and its five steps as described in Table I. The critique, or problem-finding, phase was organized around seven cards, each containing a question about the five steps in the CIP process, theoretically based on SDM. The cards were designed in such a way as to leave room for writing and sketching/drawing. The questions on the cards are given in Table II.

**Table II.** Questions to participants.

Stage	Questions
1. Introduction	1. How does the film about CIPs correspond to your own experience of CIPs? What are the differences?
2. Choices & options talk	*User questions*: 2. How am I allowed to express my needs and wishes? 3. How do I obtain knowledge about available alternatives? 4. How do I gain an understanding of the caregivers’ views on my problems and needs? *Caregiver questions*: 2. How do you go about finding out a client’s problems, needs and wishes? 3. How do I gain knowledge about a client’s alternatives? And how do I communicate with the client about those alternatives? 4. How do I express my knowledge concerning a client’s problems, needs and wishes?
3. Invitation to a meeting	5. What do you find problematic when you are invited to a CIP meeting?
4. Meeting	6. What problems have you experienced during a CIP meeting?
5. Follow-up	7. What are the problems with the follow-up of the CIP?

As illustrated in Table II, cards 2–4 had slightly different questions for users and caregivers based on their different roles in the CIP process. Cards 1 and 5–7 used the same questions for users and caregivers. Approximately 10 minutes were given for each card. The participants sat in groups of three to four. Initially they worked individually and were asked to first reflect on each question and make comments. Then, at the end of the problem-finding phase, a collaborative “analytical” session was held, where each group discussed and clustered the problems and issues written on the individual cards. Eventually, the groups gave each cluster a label describing the problems for easy recognition in the fantasy phase.

In the fantasy phase, the workshop participants were instructed to solve problems, look ahead, and think outside of their old ideas (i.e., old habits). In addition, they were instructed to include processes that they thought already worked well, in their solutions. The fantasy phase started with the participants working on their own, followed by them presenting their solutions to their group. The idea behind this method was to bring inspiration and encourage a creative flow, before a new round of individual brainstorming of solutions. The fantasy phase ended with all the participants writing their solutions on large sheets of paper before a concluding analytical session, which was carried out in a similar way as in the problem-finding phase.

Because of limited time, the implementation phase had to be shortened, but it did allow each individual participant to rank the top three most important problem-solution pairs. The Future Workshop ended with a short introduction of the next workshop which was planned, to be organized by the researchers.

In the user groups, there was a need for both individual reflection and joint discussions, but some users also needed writing support. In these cases, a research assistant took on the role of secretary. At the end of the workshops, the worksheets, including all the cards and post-it notes, were collected and later analysed by the research team. The analyses had to be performed before the “Present and Future Stories Workshops”, as described below, could be planned.

### Present and future stories workshops

The second set of workshops explored the results from the Future Workshops using narrative techniques (Rosson & Carroll, 2007) regarding improved CIP processes in the future. Stories play several different roles in participatory design. Muller (2007) suggests employing different ways of using stories to initiate dialogue with participants as a way of gaining knowledge on design opportunities and as a tool for designers to present future solutions to users.

**Figure 1.**
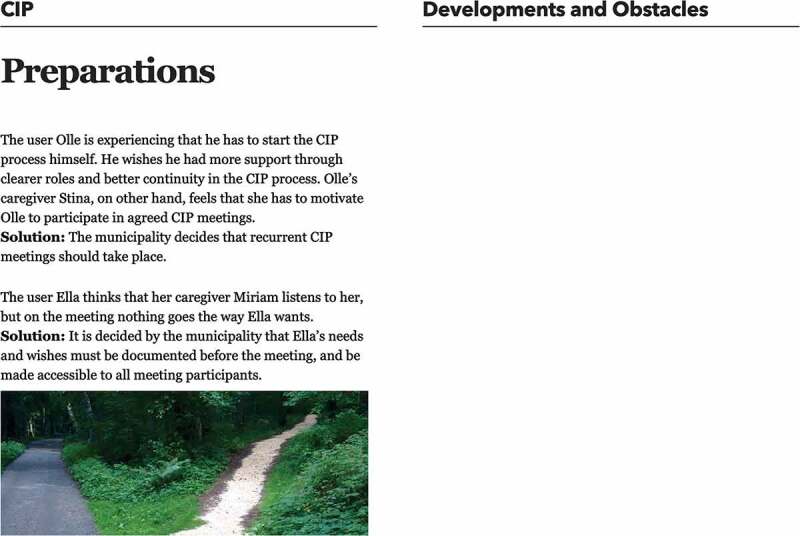
A “present and future story” as presented to the users and caregivers (translated from Swedish). On the right, the users and caregivers were to write their proposals of development, as well as the obstacles with the solutions.

In the present study, stories were created by the research team based on the results of the analysis of the problems and solutions that had emerged during the initial Future Workshops. For each phase of CIP, one or two different stories with four fictive characters, two users and their two caregivers, were created. In Figure 1, the story for the Introductory stage of the CIP is presented. All stories are presented in Appendix.

Each story was printed at the top of a blank sheet of paper on which the workshop participants were asked to describe or draw possible developments and obstacles or identify problems in the story. The purpose was to further concretize the CIP process and highlight opportunities for increased participation and SDM, but also to clarify individual and contextual barriers to this process (Kensing & Greenbaum, 2013).

During the presentation, the user workshop participants spontaneously commented on the results. Their comments were written down by a research assistant and were considered valuable knowledge for the continued development work. Moreover, as in the earlier Future Workshops, in the user groups, there was a desire for both individual reflection and joint discussion, but they also needed writing support. In these cases, a research assistant took notes.

Each workshop started with a presentation of the problems identified during the Future Workshop. A condensed version of the problems and solutions in each step of the CIP process was presented to the participants as stories. The user problems and solutions that had been identified were presented alongside the problems and solutions identified by the caregiver participants to illustrate similarities and differences. The stories were then handed out on paper, to serve as reference and make notes while the workshop leader presented them.

The participants sat in groups of three to four and when they went through each “story”, they were each asked to first quietly read the story and make comments on the pros and cons of the solutions presented. Thereafter, the participants were grouped in pairs to discuss both the stories and their own comments, followed by a short individual activity where they noted down points that had emerged during the discussion. The last step of the workshop was aimed at developing a future story for CIPs, where each group was able to further develop one of the five stages of the CIP process. The groups were instructed to use stories, storyboards, sketches or post-it notes to develop the CIP stage the group had chosen to work with. At the end of the workshop, all the worksheets were collected for further analysis by the research team.

### Setting and population

The workshops were carried out in two municipalities in Sweden. The participants consisted of caregivers and users of housing support and supported housing for people with mental disabilities. Caregivers had reported interest in participating in the study in connection with an information meeting that also included a lecture on SDM and individual coordinated plans. The caregivers had then recruited users from the services in which they worked. The inclusion criteria for participation for caregivers were working in a service that regularly used CIPs, and personal experience of carrying out such plans. The inclusion criteria for the users were own experience of mental illness, and personal experience of participating in at least one CIP process. Participating in, or having a, CIP implicitly meant that the users received care and services from both the social service and outpatient psychiatry services. In addition, a large proportion of the participating users had an addiction and contact with addiction care. All participants signed an informed consent for participation before the first workshop started.

In total, four separated groups were created (Groups I–IV in Table III), two groups of users and two groups of caregivers. The workshops were user-only or caregiver-only. Each group participated in a Future Workshop, and 1 month later in a Present and Future Stories Workshop. In total, 8 workshops were held, two for each group. Table III illustrates the participants in each workshop.

**Table III.** Workshop participants in the eight workshops.

Groups	Future Workshops	Present and Future Stories Workshops
I: Users	5 (1 woman, 4 men)	3* (1 woman, 2 men)
II: Users	4 (4 women)	1 (1 woman)
III: Caregivers	10 (6 women, 4 men)	8 (5 women, 3 men)
IV: Caregivers	7 (6 women, 1 man)	6 (5 women, 1 man)

*For this workshop, a new group of users was recruited by caregivers.

### Data analysis

All problems, solutions, developments and obstacles written down on cards and post-it notes and all stories by users and caregivers were transcribed, analysed and thematically sorted by the researchers. Workshops with users and workshops with caregivers were analysed separately.

The data were analysed in two iterations because of the constraint of having different target audiences for the results. The fact that the second (Present and Future Stories) workshop built on the outcome of the first (Future Workshop) also made things more complex, and there was a need to work with both problems and solutions during the two workshops (see below for a summary of the data collection and analysis procedure). When presenting the results from the Future Workshop in the Present and Future Stories Workshop, we aimed for recognition by the audience—in other words, we wanted both users and caregivers to recognize and acknowledge each other when they received the results during the Present and Future Stories Workshop. In a second analytical iteration of the data, groups of messages were created, inspired by the Affinity Diagram technique (Holtzblatt & Beyer, 2017). Affinity Diagrams include three different levels of themes, the main facts and observations about which are captured through written labels on coloured post-it notes. This analytical technique has several similarities with thematic analysis (cf. Braun & Clarke, 2006). The first thematic level includes the messages from the participants themselves, although because they are grouped and labelled there is no need to read the individual messages. The second level includes groups of first-level themes, giving the reader of the diagram the main findings from the data, The third level communicates more or less the conclusions you can draw from the analysis. In our analysis, we used only two analytical levels because of the condensed nature of the messages, and that the data set contained a limited number of messages compared to more field study approaches, which affinity diagramming was designed for originally. For labelling the groups of messages, Holtzblatt and Beyer (2017) propose using “I” language in order to always be as close as possible to the respondents’ statements that have been analysed. In this study, we have used “I” language for users and “we” language for caregivers.

The procedure of data collection and analysis of this study can be summarized as follows:

1. Collecting data in four Future Workshops.
2. Analysing the data from the Future Workshops.
3. Creating stories based on the results from the Future Workshops, and then using the stories in the Present and Future Stories Workshops.
4. Collecting new data in four Present and Future Stories Workshops.
5. Analysing data from all eight workshops in order to present the results, below.

## Results

The eight workshops generated rich materials illustrating challenges that users and caregivers have experienced with CIP work, as well as a large variety of solutions to these challenges. The results of the analysis are presented below, broken down into (1) challenges with CIP, followed by (2) suggestions for solutions.

### Challenges experienced with coordinated individual plans

A diagram of the themed challenges is presented below (Table IV), and will be further presented below.

**Table IV.** Themed challenges from the workshops.

Users		Caregivers	
Theme	Sub-themes	Themes	Sub-themes
**I want to be competent, but my influence is limited, and there’s a lack of support**	I want to succeed as a competent user but the caregivers demand too much of me My agency is limited My support person and I have limited power	**We don’t trust the users’ competence and they don’t give us their consent**	We’re uncertain of the users’ competence We have problems with user consents
**They talk to me, but about the wrong things**	The caregiver’s knowledge of me is limited They talk to me	**We collaborate with colleagues, and then we talk to the users**	We care about the users We talk to the users We collaborate with our colleagues We have collaboration problems with the users
**I feel responsible for something I have no control over**	I feel responsible for the CIP process I don’t prepare for the CIP meetings	**Our organization has problems with CIP work, because of a high workload, lack of documentation and poor management**	We’re stressed We don’t take notes the way we should We don’t follow the CIP process as we should
**My needs and wishes are not met, and there’s nothing after the CIP**	I don’t get anything after the CIP It takes a long time for me to get help I’m not given any options I don’t see a regular use of CIPs		

### The user’s competence

The users expressed a desire to participate in the process and to be competent, but felt that their agency was limited. They emphasized lack of trust and understanding on the part of the caregivers in relation to users’ fluctuating health and ability. When the story was about problems with initiating and inviting the user and relevant caregivers (and carers) to a CIP meeting, the users were clear about the need to establish trust-based cooperation between users and caregivers. Several users expressed how near impossible it was to conduct a CIP meeting without it being based on the users’ needs and experiences. The caregivers, on the other hand, claimed that the users were not interested in participating in the CIP process and often did not give their consent. Caregivers also expressed a low level of confidence in the users’ ability to participate in a CIP process due to their situation. Some caregivers were concerned about what they referred to as the “mental state” of the users before and during a CIP meeting, and that they might suddenly withdraw their consent, disabling the entire process.

#### Users feel responsibility but lack of control

Another challenge emphasized by both users and caregivers concerned responsibility; some caregivers demanded that users take responsibility in terms of participation and consent, but also highlighted their organizational problems with CIPs due to a heavy workload, lack of documentation and poor management. Some users, on the other hand, felt that they were largely responsible for the implementation of their CIPs and had to be in charge without support from caregivers. They felt responsible for a process that they, in reality, were unable to control.

#### The difficulties of collaboration

During the period of investigation and planning (phase 2 of CIP), several points were highlighted and problems emphasized. It became obvious that caregivers care for the users and work for the good of the users. However, though the *preferences* of clients become important clues in a process of reaching joint solutions, this work does not necessarily include *collaboration*. Instead, caregivers describe themselves as solution-focused, where possible alternatives and solutions are mainly discussed with other colleagues.

This lack of collaboration was also voiced by the users who mentioned the lack of knowledge of caregivers about users’ needs and wishes. According to the users, caregivers do not ask for the users’ knowledge; instead, they discuss “the wrong things” with the users. Wrong things in this context include how a user is doing in their home and with everyday chores. However, the perceived lack of knowledge of caregivers also comprised low awareness, according to the users, among caregivers regarding services and activities in their own practice and/within their own organization as well as services organized by other authorities.

#### The user has no say during coordinated individual plan meetings

The users’ problem identification also highlighted issues to do with power and hierarchy in relation to the different parties involved in the CIP process’. Even where users and/or their support person had created a good plan together to bring up at the CIP meeting, the plan was not always taken seriously at that meeting or was more or less ignored. Support workers—the people who spend most time with the users—were perceived as having limited power and influence at CIP meetings in relation to social workers and psychiatric nurses. This means that those who are intended to strengthen the voice of the users have not authority to do it. The users also described situations where caregivers brought suggestions for interventions to a meeting that had not been presented to the users beforehand/in advance. This situation meant that the user was not prepared, or well informed about alternatives, and had little control over the agenda of the meeting. Moreover, some users felt that caregivers made certain caregiver efforts conditional, for example, to the users agreeing to interventions they themselves felt doubtful about or that placed too high demands on them. Such perceived lack of knowledge among caregivers can have consequences on how well the proposed interventions match the needs of the users. The users described how their needs and wishes were not met, which undermined the incentive for participating in a CIP process.

### Suggested solutions

A number of solutions emerged during the fantasy phase in the Future Workshops, and when asked to cluster the solutions in the implementation phase the participants selected the most relevant solutions. These suggestions were further improved in the Present and Future Stories Workshops and further suggestions were also put forward.

#### A clear agenda for coordinated individual plans

All the participants proposed a clear agenda for the CIP process, so that it was clear what was to be discussed, who was to lead the meeting and who was to document the meeting. The caregivers suggested a number of technical solutions for how this could be done and emphasized that the meeting should have a “user perspective”, meaning that the users’ needs should be at the centre of the discussions. The users, on the other hand, suggested that the invitation should include the agenda and reflect the choices and options talk (stage 2 of creating a CIP, see Table I) between the user and the caregiver(s).

#### Trust, motivation and flexibility

One area where users’ and caregivers’ responses clearly differed concerned solutions to enhance user confidence in planning, decision-making and follow-up. The users said they wanted to trust the caregivers and be confident about their flexibility and professional knowledge. The caregivers, on the other hand, emphasized the importance of motivational work to enhance user motivation to participate in a CIP process. They suggested increasing user knowledge about the function of CIPs and how a CIP meeting could give users the opportunity to influence or change their situation. For the users, there was no perceived need to understand the importance of CIPs, but, rather, there needed to be agreement on the requirements.

Several users described the feeling of being forced into planning and mentioned possible consequences if a user missed an agreed time/appointment or was generally in poor condition during the planning process or meeting. They described how a user’s health fluctuates, and said that this could be difficult for caregivers to discern, but could result in users passively agreeing to proposed efforts, without being given the option or choice to talk, or to end or avoid the planning process and the meeting. In the Present and Future Stories Workshop, there was an increased consistency in the solutions proposed between users and caregivers in response to these experiences. One such solution was flexibility in preparation, meetings and support of users in relation to their cognitive needs and personal preferences. Table V presents the solutions proposed during the workshops.

**Table V.** Outcomes of the workshops: solutions proposed by the participants.

Story about:	Users		Caregivers	
	Themes	Sub-themes	Themes	Sub-themes
Preparation	**I see flexibility and knowledge in caregivers and others, and feel comfort from them**	Demands for confidence in caregivers A need for flexibility based on my condition and needs Improved cooperation from caregivers	**We develop and improve knowledge about CIPs and motivate the user to participate**	We develop our own and the users’ knowledge about CIPs (purpose; chairperson and secretary, and other formalities) We motivate user participation and consent
Choice & options talk	**The caregivers and I exchange knowledge**	Improved knowledge on services/alternatives among caregivers Caregivers consider my preferences during our exchange of knowledge	**We are clear and open in our provision of information to the user**	Caregivers need to show willingness to discuss alternatives, and inform the user about advantages and disadvantages Individual conditions for receiving information need to be considered
Invitation to meeting	**The invitation reflects the preparation**	My needs are central in terms of agenda, roles and pace	**The invitation is digital and based on user participation**	Digital suggestions on how to produce an invitation Clear agenda SDM training for caregivers
Meeting	**The meeting is guided by my needs and I feel in control**	My needs, in terms of location, support and company, are taken into account No care efforts are conditional Meeting documentation and summary are visible to me Someone I trust is present	**We follow a clear structure during the meeting**	Clear agenda concerning who does what Not too many items Follow-up should be scheduled It is important that there should be a meeting summary
Follow-up	**The follow-up is clear and I do not feel pressured**	Clear time plan The CIP is available to all involved persons including me Flexibility around caregivers’ expectations of me	**We decide a follow-up time and user feedback is requested**	We create a clear time plan User feedback on the meeting

#### The need for a trusted support person

One suggestion put forward by the users to enhance their comfort was that caregivers should ask them whom they would like to involve in the CIP process. The users said that the choice of a support person was sometimes a problem, and highlighted how important it is that the user is given the opportunity to decide whom they wish to invite as a support person during a meeting, instead of such a person being assigned by caregivers. Several users described being at a constant disadvantage in relation to caregivers who caused them distress. They requested a person whom they trusted and who they clearly knew would stand up for them and would be on their side.

#### Genuine collaboration on knowledge exchange

As regards solutions for enhancing the exchange of knowledge, the caregivers suggested improved collaboration with the users. They emphasized the need to improve caregivers’ attitudes towards the users and make them more interested in the views of the users, but also to encourage them to “dare to discuss possible alternatives” with the users. Daring to discuss alternatives with the users implies seriously listening to the alternatives put forward by the users, but also discussing pre-existing alternatives, regardless of whether the caregivers themselves consider a particular alternative to be the most appropriate. Some caregivers suggested a specific worksheet in the case management method as a tool to formulate user preferences and goals.

The users, on the other hand, discussed the need to improve caregivers’ knowledge of alternatives outside their own unit. In their experience, it was often the users who knew more about alternatives, and who informed caregivers of them. Therefore, they proposed that it was important to enhance the knowledge of the caregivers concerning possible schemes/activities and support outside their own organization.

#### Flexible meetings with open documentation

When the story was about *the CIP meeting* itself, there was a lively discussion in all the groups. The users argued that CIP meetings were stressful, and highlighted the complexity of wanting to show competence and agency during the meeting, while at the same time feeling unwell and having difficulty following the discussion. The users did not wish to address all their shortcomings in front of all the caregivers present at the meeting, but at the same time, it was crucial that these deficiencies were brought up in order to ensure adequate support. The users suggested a friendly conversational climate, focusing on both user needs and assets, the presence of people the users trusted, as well as efforts/actions that were not perceived as conditional. They suggested having “greater flexibility during the meeting”, by which they referred to the comprehensibility of the discussions, opportunities for a break, and documentation being put on a screen that everybody in the room is able to follow and agree on. Involving users in the documentation process implies ensuring that the user recognizes the situation described in the documentation and avoiding documentation produced from only one angle. The caregivers emphasized the need for clarity in topics to discuss, a clear structure to be held during the meeting and routines for follow up.

#### User-centred agenda and debriefings

To avoid the risk that caregivers dominate the meeting and that they add what they consider urgent issues to the agenda, caregivers expressed a need for a reasonable agenda with only a few items, all of which are important from the point of view of the user. Caregivers emphasized the need for a clear agenda that is distributed in advance, and then adhered to in the meeting. They also suggested that the final item on the agenda should be to schedule a follow-up meeting. Just like the users, the caregivers suggested that the meeting should be summed up at the end and that the documentation be distributed to everyone present. Both users and caregivers suggested that after the meeting, the user and the contact person should discuss how they had experienced the meeting and check whether there was anything the user had not understood. Such a debriefing after, instead of during, the meeting was a good way of dealing with the issue that, when asked during a meeting, most users just say that the meeting is “good” because they are in an inferior position to all the professionals present.

#### A clear, timely and flexible follow-up

Discussing solutions for a follow-up plan, and in order to ensure that users and caregivers adhered to the CIP and what had been decided at the meeting, both users and caregivers emphasized the need for a clear and timely follow-up. This assumes that the CIP is accessible to all involved; in this context, the users emphasized the impact of caregivers leaving or being on vacation, and how important it was that new/substitute caregivers were adequately informed in order to avoid disruptions in the planned care. Another suggestion from the users was flexibility in the CIP plan in terms of caregivers’ expectations of the user. The users sometimes felt pressured if expectations were too high, or felt that poor self-confidence might stand in the way of achieving highly set goals. With more flexibility regarding expectations, the dichotomy of success or failure might be eliminated.

## Discussion

The results of the participatory workshops illustrate challenges that were identified concerning the CIP process’ and a number of solutions for how these challenges might be addressed with the aim of improving user cooperation and SDM. An improved CIP process emerged from the workshops, based on the active participation of users and caregivers. The process was proposed to be familiar to and transparent for users and for caregivers from all relevant services. Coordinated individual plans reflect the needs and preferences of the user at all stages. Careful preparation of caregivers in collaboration with the user was strongly emphasized, as was the needs for flexibility in the process, as well as continuous follow-up. In addition, more specific solutions were important for the users, such as the need for a trusted support person and transparency in documentation during meetings for all participants. A condensed version of an improved CIP process is illustrated in Table VI below. Some solutions aid the whole CIP process, not only one challenge.

**Table VI.** The improved CIP process.

Stage	Content	Challenges	Solutions
1. Introduction	Creating a mutual agenda; describing roles and exploring whom to invite; and obtaining consent.	Issues with user’s competence Lack of support Problems with consent	Need for a trusted support person Caregivers need to motivate the users
2. Choice & options talk	Informed discussion about choices and treatment options.	Users and caregivers don’t collaborate	Genuine collaboration and knowledge exchange before the meeting
3. Invitation to a meeting	The meeting is to follow the agenda created by the user and caregivers.	Limited knowledge of the users and their options	The invitation reflects the collaborative preparations
4. Meeting	Decision-making skills and authority in users and caregivers: making informed decisions and creating a CIP.	Hidden and limited documentation Users’ needs and wishes are not met, or efforts are conditional	Open documentation during meetings Agreement of requirements with flexibility
5. Follow-up	Joint action plan.	There is nothing after the CIP meeting The CIP process is not followed	Debriefing after the CIP meeting A clear, timely and flexible follow-up

The workshops illustrated how caregivers focus on the user and express a genuine desire for user participation and SDM. However, they often see the user, not as a knowledge carrier, but as a person with great difficulties, which also reflects previous research (Eriksson, 2015; Sandman & Munthe, 2010). In the CIP, caregivers wish to make informed decisions together with the user, in accordance with SDM, but are unclear about how to take into account the user’s knowledge (Grim et al., 2019; Nykänen, 2019) to meet their need for involvement and influence over their own care. The process is not characterized as a collaborative process (Arnstein, 1969; Davidson et al., 2017) where users and caregivers together draw up the goals and objectives of the activities, and together make informed decisions (Elwyn et al., 2013; Treichler & Spaulding, 2017). Caregivers are to some extent aware of this challenge, and their’ solutions also focus on structure, procedures and digital solutions for improved collaboration. However, if these solutions really aim for genuine collaboration may be questioned.

The improved CIP process proposed by the users included users having access to information, and caregivers creating flexibility in the meeting based on the users’ condition and preferences, and finding ways for the users to understand what is decided and how decisions are to be followed up. In terms of Arnstein’s ladder of participation, this does not reflect collaboration, rather consultation (Arnstein, 1969) where users do not see themselves as partners but as recipients of information from caregivers. Nevertheless, there is reason to be cautious about trying to match all users to Arnstein’s model, when dealing with a complex reality (Johannesen, 2018). Users in the social services are a heterogeneous group that define influence and participation in different ways and have different perceptions and ideas about when, where and how they want collaboration and SDM (Davidson et al., 2017; Grim et al., 2019). Still, the users’ proposed CIP process illustrates an improvement in collaboration and increased user influence as the users also suggested being consulted based on their own ability.

The users’ response further illustrates a contradictory position where, on one hand, they felt a lack of competence, agency and support, and, on the other, experienced high expectations from caregivers. These expectations are also reflected in the caregivers’ suggestions for the CIP process, that the users should demand their rights—instead of the onus being on caregivers to ensure that the users’ rights are fulfilled and pave the way for this to happen in the best possible way. This ambiguity in caregivers, where the users are both considered to have a lack of ability to be involved at the same time as they are expected to express and drive their views and wishes, is also reported in other studies (Grim et al., 2019; Levin et al., 2017).

The introduction of the obligation to implement a CIP was an authority decision characterized by a top-down perspective where neither contextual nor individual factors were given any consideration. However, the purpose is for caregivers to strive for the coordination and individualization of support for people with extensive needs (SKL, Sveriges Kommuner och Landsting, 2019). The aim of this study was to explore the experiences and thoughts of users and caregivers regarding how collaboration and SDM can permeate the process of developing CIPs through a participatory design project. When individual needs, and the work and everyday life situations of both users and caregivers become the basis of a mutual learning experience concerning how a CIP process might work, the process can become a bottom-up attempt to realize CIPs (Kensing & Greenbaum, 2013). Based on Arnstein’s ladder of participation, both users and caregivers have contributed with their expertise and co-created a model for how CIPs can be implemented and how they can ensure that they include participation and SDM (Arnstein, 1969). However, designing organizational frameworks through a participatory design with vulnerable users requires flexibility and adaptation. The adjustments made in this study were that the users were offered assistance in writing and that the discussion sessions among the users were conducted partly in the full group, instead of small groups as originally planned. The users commented spontaneously and continuously on the process. This obviously affected the design of the workshop, but is considered to have provided valuable views. The workshop leaders further ensured that all the participants were involved and that their voices were heard.

A limitation of the study is that one of the user groups did not participate in the second workshop, but was replaced by caregivers with other users. One reason for this is location. The first workshop was held close to their housing, while the second was held at a site users had to travel to. This stresses the importance of conducting this type of study in locations that are easy to reach for users. Another limitation of the study concerns the recruitment of users in general. We do not know whether all users of the services were asked to participate. The study does not claim representativeness, but there is uncertainty as to whether caregivers avoided asking certain users who, for example, were considered too vulnerable to participate or people who expressed discontent with the services.

Future workshops is a participatory method for finding problems and solutions on a topic in a specific context, and in this study the method contributed to a rich data material, that differs from interviews and questionnaires, by being more creative and spontaneous using brainstorming techniques. However, it relies greatly on writing, which may imply that participants’ voices will be stronger if they have good writing skills. Moreover, future workshops starts from scratch by posing the question: What are the problems with X in the context Y. Mutual learning is an important aspect of all participatory design activities (Kensing & Greenbaum, 2013) and one could question what users and caregivers in this study learn from solely a future workshop. One conclusion is that the Present and Future stories workshop on the other hand, clearly gave the opportunity to mutual learning, by the stories that highlighted both users’ and caregivers’ behaviours, challenges and solutions. Nevertheless, without the initial Future workshops the stories had to be derived from researchers’ assumptions.

In co-creating a CIP process including increased collaboration and SDM, the participants proposed a number of solutions for CIP work that involve SDM. At the same time, the suggested CIP process exposed the need for structural changes, by making collaboration problems within the social service and between services as well as professional hierarchies visible. Based on this study and previous research (Eriksson, 2015; Nykänen, 2019), a recommendation will be to consider, at a management level, the need for improved structures for collaboration and SDM in services, in order to realize these goals.

The results from this study illustrate a CIP process that includes participation. Shared decision-making requires an active collaboration between users and caregivers, where users’ and caregivers’ knowledge and experiences are deliberated. It also requires a system that includes digital solutions, opportunities for collaboration with other authorities, and easy access to knowledge about services and evidence. Nonetheless, most important is that the participating caregivers begin to see the users as knowledge carriers, as individuals that not only is provided with information, but partners to collaborate with. Knowledge is still limited on how collaboration between users and caregivers can be promoted in connection with the development of CIPs. This study illustrate the complexity in the process and the needs for an in-depth knowledge both from a user and caregiver perspective on the conditions to manoeuvre in these participatory processes. The results also illustrate the need for increased knowledge of how organizational factors can facilitate collaboration, as well as the potential of digital tools to facilitate collaborative meetings between users and caregivers in the social services.

## Appendix. Stories of Challenges and Solutions

### Preparations

The user Olle is experiencing that he has to start the CIP process himself. He wishes he had more support through clearer roles and better continuity in the CIP process. Olle’s caregiver Stina, on other hand, feels that she has to motivate Olle to participate in agreed CIP meetings.  **Solution**: The municipality decides that recurrent CIP meetings should take place.

The user Ella thinks that her caregiver Miriam listens to her, but on the meeting nothing goes the way Ella wants.  **Solution**: It is decided by the municipality that Ella’s needs and wishes must be documented before the meeting, and be made accessible to all meeting participants.

### Knowledge exchange

Olle thinks that there is no discussion about his problems and needs which includes his point of view. He also feels that the caregivers don’t tell him how they look at his problems and needs.
**Solution**: The caregivers take the decision that Olle and Stina should have a meeting where they together discuss and formulate Olle’s needs and wishes.

The caregivers includes Ella in the search for care alternatives although it requires more of the caregivers to include Ella’s view in this process. Ella’s personal contact Miriam communicates with other units about which possible solutions that exist for Ella.
**Solution**: When there are several alternative solutions for Ella, Miriam will go through them with Ella well in advance before the upcoming CIP meeting. The alternatives are also documented and accessible for Ella—she could read them whenever she wants.

### Invitation to meeting

Olle and Ella experience that the caregivers from different units are badly prepared. The invitation to meeting lacks a clear agenda, and who is going to lead the meeting and being the secretary is also unclear.
**Solution 1**: The CIP developers propose that an invitation to meeting is sent out well before the meeting. Because the CIP meetings now are regular, the unit’s computer systems could easily send out reminders about the necessity to set up a new CIP meeting.
**Solution 2**: In the invitation to meeting the user’s view on her needs and wishes (which she has written together with her contact person) are available to all parties to read. In the same way, the caregivers proposals of different alternatives available to the user and the caregivers that are invited to the meeting.

### The meeting

Olle is worried about the CIP meeting.
**Solution**: Stina tells Olle that they are going to have a pre-meeting before the CIP meeting, where they could prepare, and go through Olle’s different alternatives.

Stina and Olle experience that the CIP meeting itself is messy, and that the meeting quickly loses focus on Olle’s needs and wishes. The meeting is stressful, lacks structure, and has no clear agenda. In addition, the meeting room is not suitable for CIP meetings. Who leads the meeting, and who documents it are also unclear.
**Solution**: The CIP developers decide to development a meeting format which will give more power to the user, and counteracts power imbalance. An education for the CIP meeting will also be developed. The developer group also decides that the meetings should be held in a place where the user is comfortable, and the meeting time are set to be two hours as standard to mitigate stressful meetings.

### The follow-up

Olle and some of his friends, which also are users, are feeling the same: “We don’t know what’s happening” and “The caregivers require too much of us during the CIP meetings that we cannot fulfil.” The staff on the other hand know what will happen, but they experience that there is no system to communicate this to the clients. The caregivers are also uncertain on who will do what.
**Solution**: Caregivers and the user formulate requirements and goals together to mitigate the problem of too high expectations on the user. A follow-up meeting must be booked during the CIP meeting. The documentation from the CIP meetings should be shared with them who failed to attend the meeting, and there should be a routine for how to follow up what have been decided on during the meeting.
